# Development and validation of a clinically applicable biomarker panel for identifying autoimmune type IIb chronic spontaneous urticaria

**DOI:** 10.1016/j.waojou.2026.101466

**Published:** 2026-09-24

**Authors:** Jiang Ji, Lu Chen, Ni Ma, Yuxu Yao, Chuqiao Zhang, Enze Li, Qingqing Jiao

**Affiliations:** a Department of Dermatology, The Second Affiliated Hospital of Soochow University, Suzhou, 215004, China; b Central Research Laboratory, The First Affiliated Hospital of Soochow University, Suzhou, 215006, China; c Department of Dermatology, The First Affiliated Hospital of Soochow University, Suzhou, 215006, China

**Keywords:** Chronic spontaneous urticaria, Immunoglobulin E, Thyroid peroxidase, Autologous serum skin test, Biomarkers

## Abstract

**Background:**

Chronic spontaneous urticaria (CSU) includes 2 autoimmune endotypes, type I (autoallergic) and type IIb (autoimmune), which differ in pathogenesis and treatment response. However, practical criteria for distinguishing these endotypes in routine clinical practice remain limited.

**Objective:**

This study aimed to evaluate whether combinations of readily available clinical parameters facilitate identification of probable type IIb CSU.

**Methods:**

A total of 125 CSU patients were evaluated using 3 diagnostic combinations: (1) high IgG anti–thyroid peroxidase antibodies (IgG-anti-TPO) with low total IgE; (2) high IgG-anti-TPO with a positive autologous serum skin test (ASST+); and (3) low total IgE with ASST+. Clinical and laboratory features were compared between presumed endotypes. Serum cytokine profiles were analyzed and validated in an independent cohort of 40 patients. A predictive graphical model integrating clinical and laboratory variables was developed.

**Results:**

All 3 combinations identified patient subsets with features compatible with type IIb CSU, including trends toward higher disease activity (UAS7), peripheral eosinopenia and basopenia. The combination of high IgG-anti-TPO with ASST positivity was associated with the most comprehensive type IIb-compatible features. Cytokine profiles also differed between endotypes: serum IL-6 tended to be lower in possible type IIb CSU, whereas IL-17 A and TNF-α tended to be higher, compared with the remaining CSU patients. These patterns were directionally supported in the independent validation cohort. The predictive nomogram model demonstrated good discriminatory performance.

**Conclusions:**

Combinations of IgG-anti-TPO, total IgE, and ASST provide practical reference tools for identifying probable type IIb CSU, with high IgG-anti-TPO plus ASST positivity showing the highest clinical relevance. Distinct cytokine patterns further support endotype-related immune differences and may aid individualized management.

## Introduction

Chronic spontaneous urticaria (CSU), defined by recurrent pruritic wheals with or without angioedema persisting for more than 6 weeks, is a common and disabling disease that substantially impairs quality of life and affects performance at work and school.^1^, ^2^, ^3^, ^4^ Increasing evidence indicates that CSU comprises immunologically distinct endotypes with important implications for disease course and treatment response.^2^^,^^5^

Two major autoimmune endotypes have been proposed:^6^, ^7^, ^8^ type I (autoallergic) CSU, driven by IgE autoantibodies against autoantigens and typically associated with elevated total IgE, coexisting atopy, and favorable responses to omalizumab; and type IIb (autoimmune) CSU, mediated by IgG autoantibodies against IgE or FcεRI and frequently linked to concomitant autoimmune diseases.^9^, ^10^, ^11^, ^12^ Patients with type IIb CSU generally show higher disease activity, low total IgE, and poorer responses to antihistamines and omalizumab, but better responses to immunosuppressants such as cyclosporine.^10^^,^^13^ Other related laboratory features associated with type IIb CSU include eosinopenia, basopenia, elevated C-reactive protein, positive antinuclear antibodies, and increased thyroid autoantibodies, among others.^14^^,^^15^ Accurate endotype identification is therefore clinically relevant for prognosis and treatment selection.

At present, definitive identification of type IIb CSU requires all 3 assessments (Fig. 1A)—ASST, BAT/BHRA, and anti-FcεRI/IgE IgG autoantibody—to be simultaneously positive,^2^^,^^16^^,^^17^ which is difficult to fulfill in routine clinical practice and has not been incorporated into current international guidelines.^18^, ^19^, ^20^ Although ASST is simple and widely accessible, its specificity is limited when used alone. Combining ASST with thyroid autoantibodies and total IgE may improve identification of probable type IIb CSU.^21^^,^^22^ Notably, IgG anti-thyroid peroxidase antibodies and low total IgE have been proposed as practical surrogate markers.^23^

**Fig. 1:**
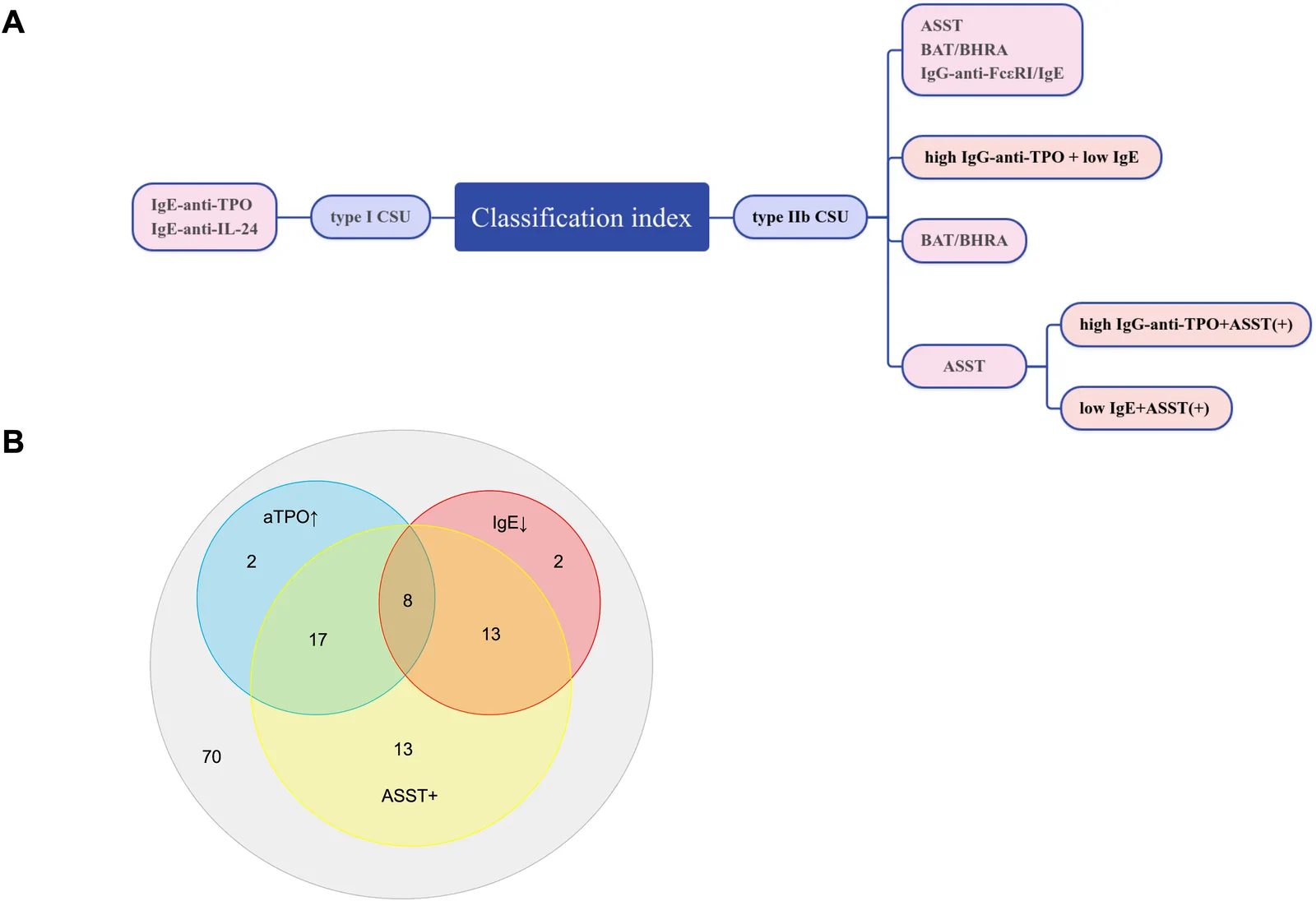
**Classification index and Venn diagram.** The figure illustrates classification index for type I and type IIb CSU (A). The diagram shows patient distribution among 3 biomarker combinations: aTPO↑+ASST+ (n = 25, 20.0%), IgE↓+ASST+ (n = 21, 16.8%), and aTPO↑+IgE↓ (n = 8, 6.4%) (B)

Despite these advances, there remains a lack of feasible, standardized screening strategies with sufficient sensitivity and specificity to identify type IIb CSU in routine care. Therefore, this study aimed to evaluate whether combinations of readily available clinical parameters—high IgG anti-TPO, low total IgE, and a positive ASST—can facilitate pragmatic identification of probable type IIb CSU and provide clinically useful guidance for endotype-oriented management.

## Methods

### Patients’ characteristics

A total of 125 chronic spontaneous urticaria (CSU) patients were recruited at the dermatology outpatient department and ward of the Second Affiliated Hospital of Soochow University from 2019 to 2022. All patients fulfilled the EAACI/GA ^2^LEN/EDF/WAO criteria for CSU diagnosis (spontaneous wheals with or without angioedema appearance daily or almost daily for ≥6 weeks, with no obvious external specific trigger). This study was approved by the Ethics Committee of The Second Affiliated Hospital of Soochow University (JD-LK-2019-105-01).

### Disease activity assessment

Disease activity was assessed with the UAS7, which sums daily wheal and pruritus scores (each 0–3) over 7 days (total 0–42), classified as well-controlled (0–6), mild (7–15), moderate (16–27), or severe (28–42).^1^ Quality of life was assessed with the DLQI (10 items, total 0–30; higher scores indicate greater impairment).^24^

### ASST

The autologous serum skin test was performed as recommended by the 2009 EAACI/GA^2^LEN task force consensus report on the autologous serum skin test in urticaria. Wheal responses were observed 30 min after testing. ASST was considered positive when the serum-induced wheal diameter was at least 1.5 mm greater than that of the negative control.^25^

### Laboratory measurements

Blood was drawn and analyzed for routine patient care. Data regarding the following laboratory tests were collected from patient records: eosinophil count; basophil count; CRP; antinuclear antibodies; total serum IgE; IgG-anti-TPO; interleukin 2,4,6,10,17 A (IL-2,4,6,10,17 A), tumor necrosis factor alpha (TNF-α), interferon gamma (IFN-γ). Cut-off values for peripheral blood eosinopenia and basopenia are ≤0.05∗10^9^/L and ≤0.01∗10^9^/L, respectively.^2^ High IgG-anti-TPO (aTPO↑) is defined as ≥34 kU/L, and low total IgE (IgE↓) is defined as ≤40 IU/ml.^23^ The critical value of CRP, ANA and cytokines were based on the reference range of laboratory indicators in the Second Affiliated Hospital of Soochow University.

### Criteria for screening

In the present study, we used 3 biomarker combinations to screen patients with type I and type IIb CSU, namely: (1) high IgG-anti-TPO and low total IgE; (2) high IgG-anti-TPO and ASST+; (3) low total IgE and ASST+.

### Verification and clinical relevance of key biomarkers

In this study, 40 CSU patients were recruited before treatment (Table 1) at the Department of Dermatology, The Second Affiliated Hospital of Soochow University for provocation testing and extensive clinical characterization. Patients were advised to stop antihistamine intake at least 3 days before any of the mentioned tests below. None of the patients had been taking topical or systemic steroids or other immunosuppressive therapy in the last 2 weeks before the tests. For further validation of our findings, the expression levels of 3 key cytokines (IL-6, TNF-α, and IL-17 A) and several other cytokines (including IL-2, IL-4, IL-10, and IFN-γ) were measured by ELISA in the serum of 40 CSU patients according to the manufacturer's protocol (R&D Systems, Minneapolis, MN, U.S.A.).

**Table 1:** Patient characteristics.

	CSU N = 40
Age (years)	38.43 ± 15.02
Height (cm)	164.80 ± 6.92
Weight (kg)	60.55 ± 9.20
Female (%)	72.50
BMI (kg/m^2^)	22.25 ± 2.71
Baseline IgE (ng/ml)	250.20 ± 265.30
Baseline UAS7	27.08 ± 8.87
Baseline DLQI	8.03 ± 3.48
Duration of disease (months)	51.73 ± 81.43

**Note**: The statistical characteristics of 40 CSU patients were described using descriptive statistics (mean and percentage)

### Nomogram prediction model

Variables with a P-value <0.1 in the univariate analysis were included in the multivariate logistic regression analysis to identify the independent risk factors for type IIb CSU. Based on the results of the multivariate analysis, the nomogram prediction model was established using the rms package in R software (version 3.4.0). The receiver operating characteristic (ROC) curve was drawn to evaluate the predictive value of the prediction model for the occurrence of type IIb CSU. Internal validation was carried out using the bootstrap method; the concordance index (C-index), a discrimination evaluation index, was calculated, and the calibration plot was drawn.^26^

### Statistical analysis

Continuous variables were described as mean ± SD or median (IQR) as appropriate and compared between groups using the Mann–Whitney *U* test; categorical variables were compared using Fisher's exact test. Correlations between continuous variables were assessed using Spearman's rank correlation coefficient. To control for multiple testing, the Benjamini–Hochberg false discovery rate (FDR) method was applied separately within 4 predefined domains: continuous cytokines, dichotomized cytokines, clinical parameters (age, UAS7, DLQI, sex), and laboratory parameters (eosinophil count, basophil count, CRP, ANA). An FDR-adjusted q < 0.05 was considered statistically significant; findings with nominal P < 0.05 that did not survive FDR correction were considered exploratory. Data were analyzed using SPSS software (version 20) and R software (version 3.4.0).

## Results

### High IgG-anti-TPO combined with ASST positivity shows the strongest association with type IIb–compatible features

Clinical characteristics and laboratory parameters of the 125 CSU patients were analyzed according to 3 different clinical combinations (Fig. 1B–Table 2). Three clinical combinations were evaluated to identify patients with features compatible with type IIb autoimmunity.

**Table 2:** Demographic, clinical features, laboratory markers and cytokines of 125 CSU patients.

Screen combination	1: aTPO↑ + IgE↓		2: aTPO↑ + ASST+		3: IgE↓ + ASST+	
	Met n = 8	Not met n = 117	Met n = 25	Not met n = 100	Met n = 21	Not met n = 104
**Clinical features**						
**Age (years), mean ± SD**	39.50 ± 12.94	39.82 ± 16.24	43.40 ± 12.65	38.88 ± 16.68	42.90 ± 15.49	39.16 ± 16.10
**Female, % (n)**	87.50% (7)	69.23% (81)	92.00% (23)	**65.00% (65)∗∗ ^†^**	80.95% (17)	68.27% (71)
**UAS7, mean ± SD**	**26.00** ± **12.01**	**17.36** ± **10.65∗**	**22.56** ± **9.84**	**16.75** ± **10.88∗ ^†^**	**22.71** ± **10.58**	**16.94** ± **10.75∗**
**DLQI, mean ± SD**	7.25 ± 3.49	6.80 ± 3.84	7.76 ± 3.49	6.60 ± 3.87	7.81 ± 3.95	6.63 ± 3.77
**Laboratory parameters, % (n)**						
**Eosinopenia**	**62.50% (5)**	**20.51% (24)∗ ^†^**	**44.00% (11)**	**18.00% (18)∗ ^†^**	**42.86% (9)**	**19.23% (20)∗ ^†^**
**Basopenia**	**75.00% (6)**	**27.35% (32)∗∗ ^†^**	**48.00% (12)**	**26.00% (26)**	**52.38% (11)**	**25.96% (27)∗ ^†^**
**Elevated CRP**	37.50% (3)	17.09% (20)	**36.00% (9)**	**14.00% (14)∗ ^†^**	28.57% (6)	16.35% (17)
**ANA positive, % (n)**	50.00% (4)	25.64% (30)	**52.00% (13)**	**24.00% (24)∗ ^†^**	**52.38% (11)**	**25.00% (26)∗ ^†^**
**Cytokines (pg/ml), median (IQR)**						
**IL-2**	2.37 (2.18)	2.35 (1.59)	1.96 (1.62)	2.48 (1.54)	2.68 (1.44)	2.30 (1.65)
**IL-4**	1.93 (4.53)	2.96 (2.89)	2.88 (2.83)	2.88 (2.95)	2.18 (2.96)	2.98 (2.93)
**IL-6**	3.25 (3.61)	4.79 (6.29)	**2.76 (2.00)**	**5.08 (6.75)∗∗∗ ^††^**	3.83 (3.87)	4.78 (6.22)
**IL-10**	3.67 (5.01)	2.51 (2.21)	2.15 (2.05)	2.57 (2.63)	3.23 (2.68)	2.53 (2.21)
**IL-17A**	**14.73 (13.24)**	**5.99 (7.54)∗∗ ^†^**	**8.79 (11.30)**	**5.65 (9.34)∗**	**8.97 (15.95)**	**5.77 (7.91)∗**
**TNF-α**	**5.86 (5.45)**	**2.41 (2.67)∗**	2.41 (4.07)	2.54 (2.61)	**5.00 (3.71)**	**2.19 (2.34)∗∗ ^††^**
**IFN-γ**	1.79 (5.36)	2.36 (2.60)	2.55 (2.50)	2.24 (2.76)	2.19 (2.92)	2.31 (2.56)
**Cytokines** elevated, **% (n)**						
**IL-2↑**	25.00% (2)	29.91% (35)	28.00% (7)	30.00% (30)	33.33% (7)	28.85% (30)
**IL-4↑**	37.50% (3)	33.33% (39)	36.00% (9)	33.00% (33)	28.57% (6)	34.62% (36)
**IL-6↑**	0.00% (0)	30.77% (36)	**4.00% (1)**	**35.00% (35)∗∗ ^††^**	19.05% (4)	30.77% (32)
**IL-10↑**	0.00% (0)	5.13% (6)	0.00% (0)	6.00% (6)	4.76% (1)	4.81% (5)
**IL-17A↑**	12.50% (1)	9.40% (11)	4.00% (1)	11.00% (11)	9.52% (2)	9.62% (10)
**TNF-α↑**	**75.00% (6)**	**29.91% (35)∗**	40.00% (10)	31.00% (31)	**61.90% (13)**	**26.92% (28)∗∗ ^†^**
**IFN-γ↑**	37.50% (3)	21.37% (25)	20.00% (5)	23.00% (23)	28.57% (6)	21.15% (22)

**Note**: Statistical significance is highlighted as bold and is indicated as ∗P < 0.05, ∗∗P < 0.01, and ∗∗∗P < 0.001 (nominal, uncorrected). **^†^**q < 0.05 and **^††^**q < 0.01 denote significance after BH-FDR correction within each predefined domain (continuous cytokines, dichotomized cytokines, clinical parameters, laboratory parameters); no symbol indicates no significant difference. Cutoffs are defined as follows: basopenia 0.01 × 10^9^/L, eosinopenia 0.05 × 10^9^/L; CRP 10 mg/L, IgG-anti-TPO 34 kU/L, IgE 40 IU/mL; IL-2 3, IL-4 3.5, IL-6 8.5, IL-10 7, IL-17 A 30, TNF-α 3.5, IFN-γ 4 pg/mL

The first combination, defined by high IgG-anti-TPO levels and low total IgE, identified 8 patients (8/125, 6.4%) as probable type IIb CSU. Compared with the remaining 117 patients, this subgroup showed significantly increased frequencies of peripheral eosinopenia (62.5% vs. 20.5%) and basopenia (75.0% vs. 27.4%), both of which remained significant after BH-FDR correction (q = 0.033 for both). They also exhibited a trend toward higher disease activity (UAS7: 26.00 ± 12.01 vs. 17.36 ± 10.65; P = 0.044). No significant differences were observed in ANA positivity, high CRP, age, sex, or DLQI.

In contrast, the second combination, integrating high IgG-anti-TPO levels with ASST positivity, classified 25 patients (25/125, 20.0%) with features consistent with type IIb CSU, while presenting the most comprehensive type IIb-associated clinical profile. Relative to the other 100 patients, this subgroup showed higher UAS7 scores (22.56 ± 9.84 vs. 16.75 ± 10.88; q = 0.024) and a female predominance (92.0% vs. 65.0%; q = 0.024). They also exhibited significantly increased frequencies of eosinopenia (44.0% vs. 18.0%; q = 0.025), CRP (36.0% vs. 14.0%; q = 0.025), and ANA positivity (52.0% vs. 24.0%; q = 0.025). Basophil counts were also lower (P = 0.038), and basopenia was more frequent (48.0% vs. 26.0%; P = 0.050); however, neither finding remained significant after BH-FDR correction (q = 0.050). No significant differences were observed in age or DLQI.

The third combination, based on low total IgE levels and ASST positivity, identified 21 patients (21/125, 16.8%) with probable type IIb CSU. Compared with the remaining 104 patients, these patients exhibited significantly increased frequencies of eosinopenia (42.9% vs. 19.2%) and basopenia (52.4% vs. 26.0%), as well as a higher proportion of ANA positivity (52.4% vs. 25.0%), all of which remained significant after BH-FDR correction (q = 0.035 for all). They also showed a trend toward higher UAS7 scores (22.71 ± 10.58 vs. 16.94 ± 10.75; P = 0.027). No significant differences were observed in CRP, age, sex, or DLQI.

Collectively, all 3 combinations identified patient subsets sharing core features compatible with type IIb, while the combination of high IgG-anti-TPO levels and ASST positivity demonstrated the strongest association with features consistent with type IIb CSU. However, the combined assessment of high anti-TPO IgG and low total IgE is the simplest to perform and shows the highest feasibility in clinical practice.

### Type IIb CSU may be characterized by a distinct cytokine profile

To determine whether the 3 clinical combinations revealed potential immunological differences, serum cytokine profiles were compared between the different patient groups (Table 2).

Patients classified as probable type IIb CSU by the first combination (high IgG-anti-TPO and low total IgE) exhibited significantly higher serum IL-17 A levels (median 14.73 vs. 5.99 pg/mL; q = 0.043 after FDR correction). Serum TNF-α levels also tended to be higher (median 5.86 vs. 2.41 pg/mL; P = 0.021), as did the proportion of patients with elevated TNF-α (≥3.5 pg/mL; 75.0% vs. 29.9%; P = 0.015); however, these TNF-α findings did not survive FDR correction.

Similarly, when the second combination (high IgG-anti-TPO and ASST positivity) was applied, probable type IIb CSU patients showed a marked reduction in serum IL-6 levels (median 2.76 vs. 5.08 pg/mL; q = 0.002 after FDR correction) and a lower frequency of elevated IL-6 (≥8.5 pg/mL; 4.0% vs. 35.0%; q = 0.009). Serum IL-17 A levels also tended to be higher (median 8.79 vs. 5.65 pg/mL; P = 0.020), although this difference did not survive FDR correction.

Consistent with these findings, patients identified by the third combination (low total IgE and ASST positivity) displayed significantly elevated serum TNF-α levels (median 5.00 vs. 2.19 pg/mL; q = 0.007 after FDR correction) and a higher proportion of TNF-α–high individuals (61.9% vs. 26.9%; q = 0.028). Serum IL-17 A levels also tended to be higher (median 8.97 vs. 5.77 pg/mL; P = 0.036), although this difference did not survive FDR correction.

Taken together, despite differences in classification standards, all 3 combinations converged on a shared cytokine pattern in probable type IIb CSU, characterized by lower IL-6 and higher IL-17 A and TNF-α expression, supporting a distinctive inflammatory endotype.

### Validation cohort supports distinct cytokine patterns in probable type IIb CSU

To validate these cytokine patterns, serum cytokines and clinical parameters were analyzed in an independent cohort of 40 CSU patients (Table 1, Fig. 2).

**Fig. 2:**
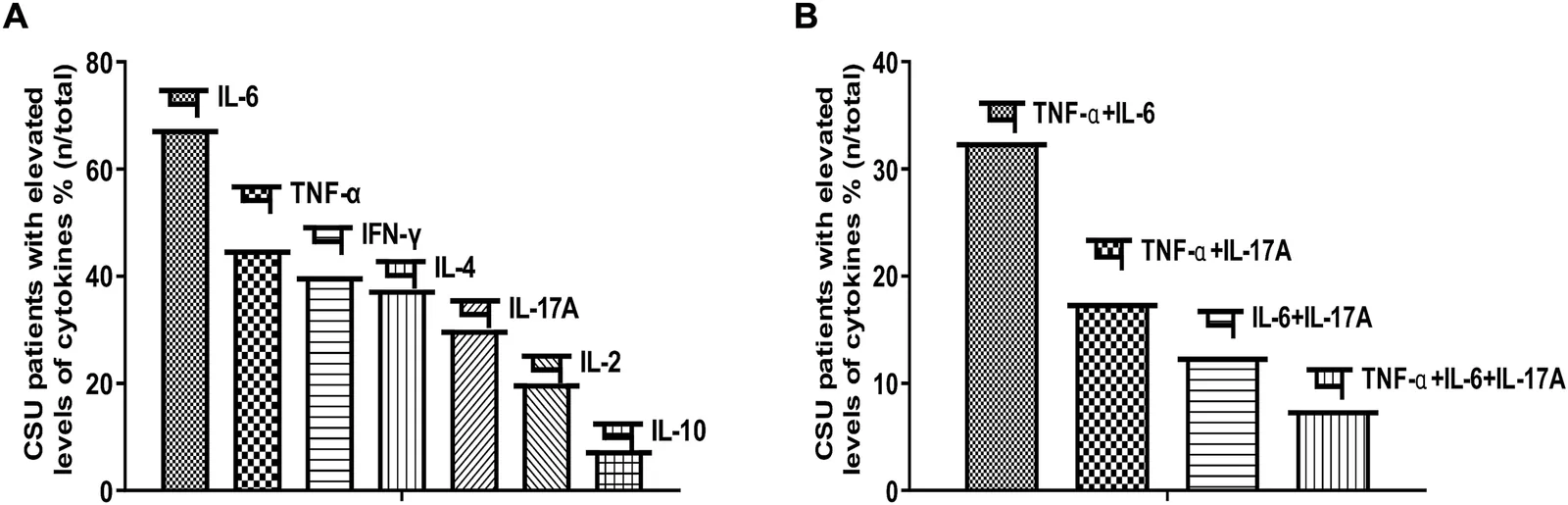
**The levels of several cytokines in CSU patients.** The levels of IL-2, IL-4, IL-6, IL-10, IL-17 A, TNF-α, and IFN-γ in the serum of patients with CSU (N = 40). Elevated levels of a single cytokine (A) and 2 cytokine combinations (B) in CSU patients

Elevated serum IL-6 (≥8.5 pg/mL), TNF-α (≥3.5 pg/mL), and IL-17 A (≥30 pg/mL) were observed in 67.5%, 45.0%, and 30.0% of patients, respectively (Fig. 2A). Co-elevation of 2 cytokines occurred frequently, most commonly IL-6 with TNF-α (32.5%), followed by TNF-α with IL-17 A (17.5%) and IL-6 with IL-17 A (12.5%) (Fig. 2B).

Correlation analyses demonstrated a positive association between serum IL-6 and total IgE levels (r = 0.376, P = 0.017; Fig. 3A–C). Patients with elevated IL-6 had significantly higher total IgE levels, and vice versa. In contrast, ASST-positive patients exhibited significantly lower IL-6 levels compared with ASST-negative patients (P = 0.045; Fig. 3J).

**Fig. 3:**
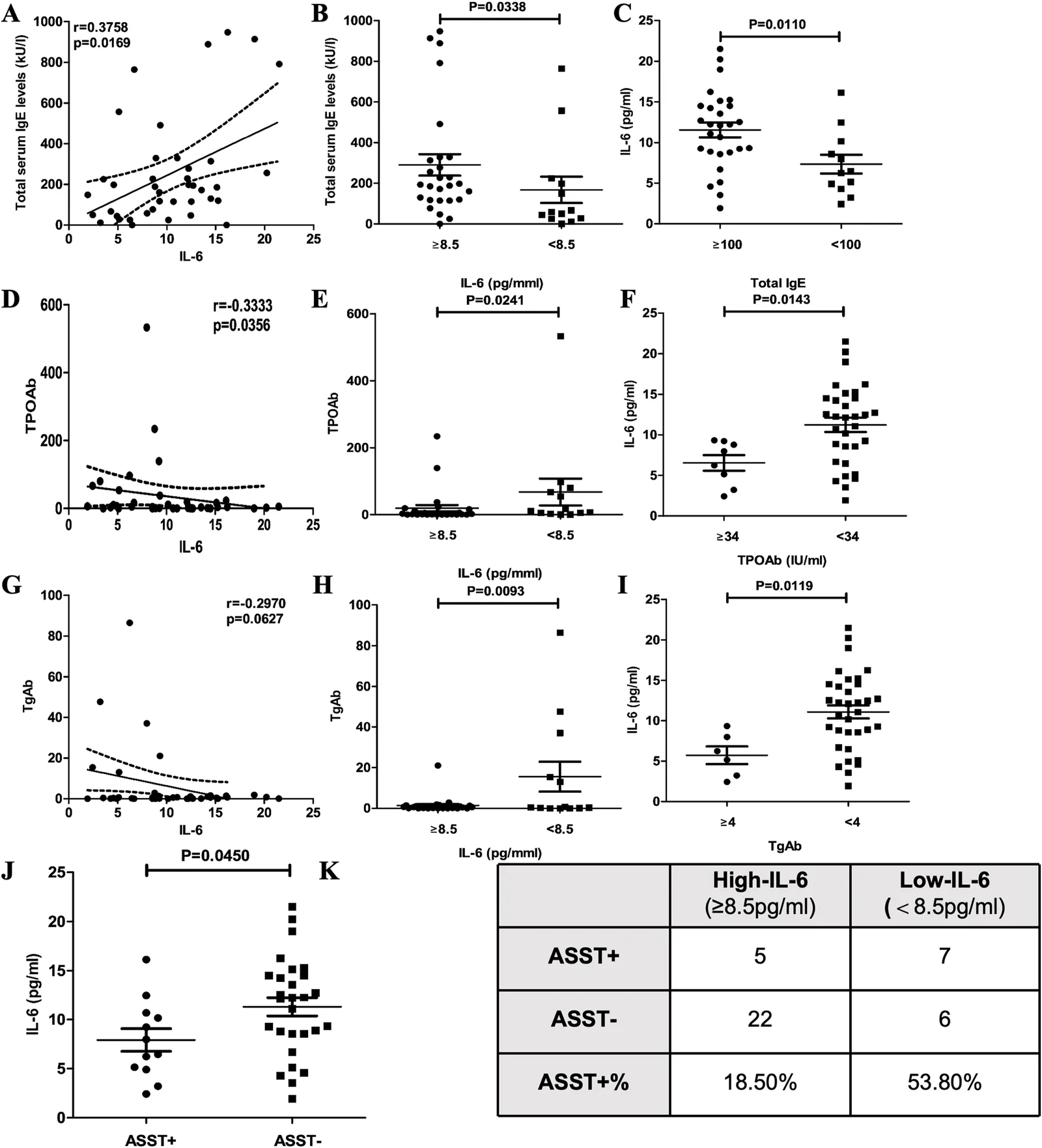
**Association between serum IL-6 level and clinical or laboratory features of autoimmune CSU.** CSU patients were divided into 2 groups based on IL-6 level or total IgE level. The correlation between serum IL-6 level and total IgE level in serum (A–C), serum IL-6 level with ASST in CSU (J, K), and serum IL-6 level with TgAb level in CSU (G–I). The association of IL-6 level with TPOAb level in serum (D–F)

Furthermore, serum IL-6 levels showed negative correlations with thyroid autoantibodies, including TPOAb (r = −0.333, P = 0.036) and TgAb (r = −0.297, P = 0.063). Patients with low IL-6 levels displayed significantly higher TPOAb and TgAb levels, whereas patients with low thyroid antibody levels exhibited higher IL-6 concentrations (Fig. 3D–I).

Weak inverse trends were observed between TNF-α and total IgE or TPOAb, although these did not reach statistical significance (Supplementary Fig. 1). Correlation analysis further revealed a positive association between TNF-α and IL-17 A levels (Table 3).

**Table 3:** Cytokines of 40 CSU patients.

Correlation coefficients (P value)	IL-6	TNF-α	IFN-γ	IL-4	IL-17 A	IL-2	IL-10
**IL-6**		0.1043 (0.5218)	−0.04923 (0.7629)	−0.08035 (0.6221)	−0.1244 (0.4443)	−0.1788 (0.2697)	0.05734 (0.7252)
**TNF-α**			0.1932 (0.2323)	0.1818 (0.2615)	**0.3654 (0.0204)∗**	0.2685 (0.0939)	0.1826 (0.2593)
**IFN-γ**				**0.3346 (0.0349)∗**	0.1687 (0.2981)	**0.4527 (0.0034)∗∗**	**0.6275 (< 0.0001)∗∗∗**
**IL-4**					−0.06402 (0.6947)	**0.4693 (0.0023)∗∗**	**0.3435 (0.0300)∗**
**IL-17A**						−0.05257 (0.7473)	0.1409 (0.3859)
**IL-2**							0.1528 (0.3465)
**IL-10**							

**Note**: Statistical significance of correlations is highlighted as bold and is indicated as ∗∗∗P < 0.001, ∗∗P < 0.01, and ∗P < 0.05, no asterisk indicates no significance

### A clinically applicable nomogram model supports identification of probable type IIb CSU

Based on the distinct clinical and immunological features observed in type IIb CSU, a nomogram prediction model was developed to estimate the probability of this endotype in individual patients (Fig. 4A).

**Fig. 4:**
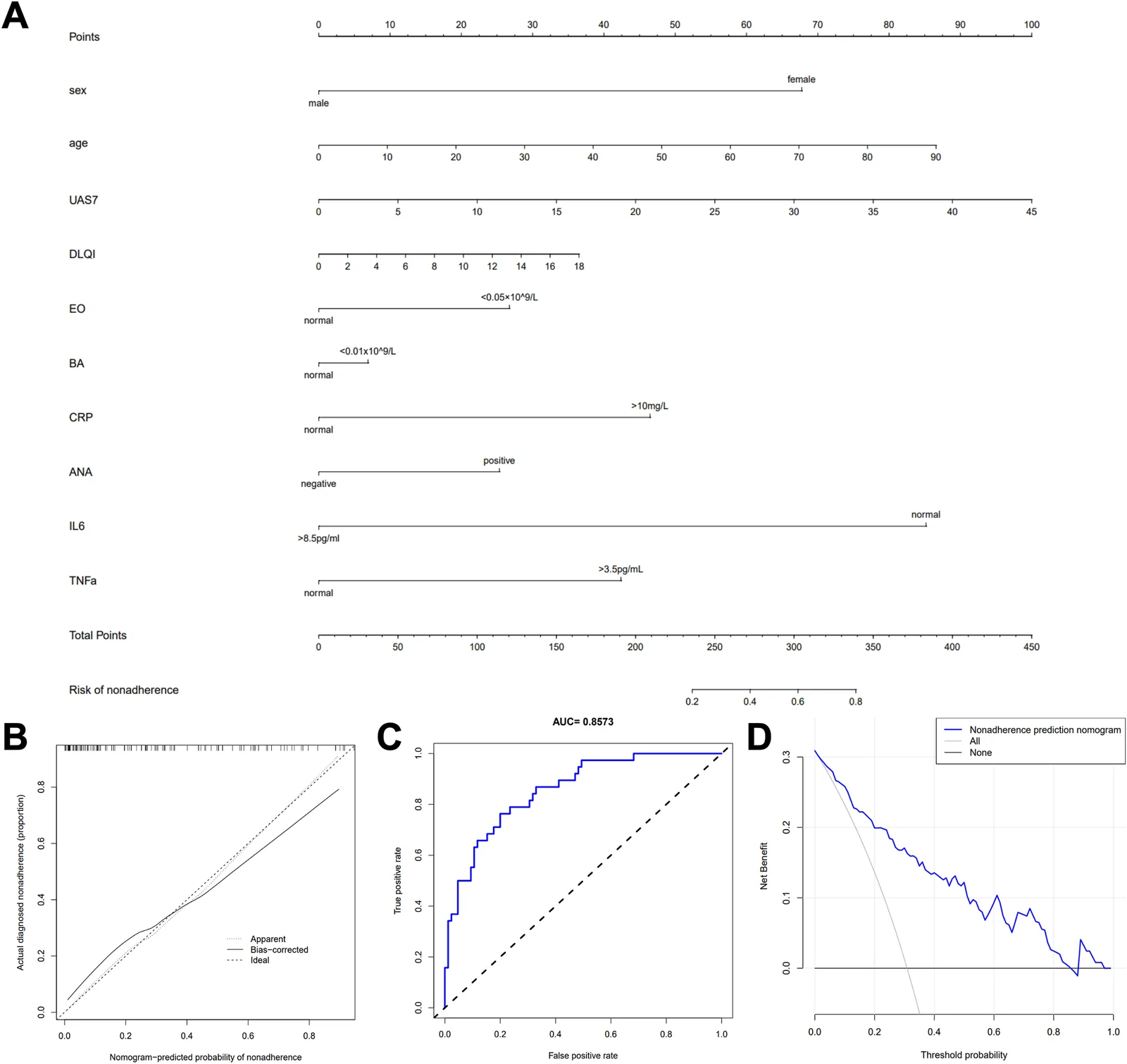
**The nomogram prediction model forprobabletype IIb CSU patients**. Individualized nomogram prediction models for type IIb risk in CSU patients (A). Calibration curves of the prediction model in the modeling and validation populations (B). ROC of the prediction model in the modeling and validation populations (C). Decision curve of the prediction model in the modeling and validation populations (D)

To ensure that the model provided independent predictive information, variables used for endotype classification in the primary analyses (total IgE, IgG-anti-TPO, and ASST) were intentionally excluded. The final model incorporated 10 routinely available clinical and laboratory parameters, including age, UAS7, DLQI, sex, peripheral eosinophil and basophil counts, C-reactive protein, antinuclear antibody status, serum IL-6, and serum TNF-α.

Each variable was assigned a weighted score in the nomogram, allowing the cumulative score to correspond to an estimated probability of type IIb CSU. Continuous variables (age, UAS7, and DLQI) contributed proportionally according to their measured values, whereas categorical variables were dichotomized based on clinically relevant cut-off values.

Model performance was assessed using calibration, discrimination, and clinical utility analyses. The calibration curve demonstrated good agreement between predicted and observed probabilities (Fig. 4B). Discriminatory ability was high, with an area under the receiver operating characteristic curve of 0.857 (Fig. 4C). Decision curve analysis further indicated that the model provided a net clinical benefit across a wide range of threshold probabilities (Fig. 4D).

Collectively, this nomogram model integrates clinical severity, autoimmune markers, inflammatory indicators, and cytokine signals to provide a practical tool for supporting the identification of probable type IIb CSU in routine clinical settings.

## Discussion

The present study aimed to facilitate rapid identification of probable type IIb CSU using combinations of clinically accessible biomarkers, thereby supporting treatment selection and prognosis assessment in routine practice. Based on previous evidence, IgG anti–thyroid peroxidase antibodies (IgG-anti-TPO), total IgE, and the autologous serum skin test (ASST) were selected as key indicators due to their availability and established associations with autoimmune CSU.^10^^,^^22^^,^^23^^,^^27^ Compared with functional assays such as the basophil activation test and IgG anti-FcεRI/IgE, which require specialized equipment, these parameters can be readily implemented in most clinical settings. IgG-anti-TPO was prioritized over other thyroid autoantibodies given its higher prevalence in CSU and its association with autoimmune comorbidity and poor response to omalizumab.^28^^,^^29^

### Diagnostic value of different biomarker combinations

Using 3 combinations of IgG-anti-TPO, total IgE, and ASST, we evaluated their ability to identify patients with clinical features consistent with type IIb CSU.^22^^,^^30^^,^^31^ The combination of high IgG-anti-TPO with ASST positivity identified the largest subset of patients and captured the most comprehensive features consistent with type IIb autoimmunity, suggesting higher sensitivity and reference value. In contrast, the combination of high IgG-anti-TPO with low total IgE demonstrated greater feasibility for routine clinical application, as both parameters are widely available without the need for in vivo testing. Importantly, the proportions and clinical characteristics of patients identified by these combinations were consistent with previous reports, supporting the reliability of this pragmatic screening strategy.^16^^,^^22^^,^^23^ In addition, our team's previous related work showed that patients with differing responses to omalizumab also exhibited corresponding differences in several laboratory parameters, suggesting that these biomarkers may reflect the underlying disease endotype.^32^ However, no single biomarker proved sufficiently accurate to predict response to omalizumab.^33^ The present study therefore aimed to evaluate whether a panel of combined biomarkers could enhance the predictive accuracy for probable type IIb CSU.

### Distinct cytokine profiles in CSU endotypes

Accumulating evidence indicates that CSU comprises immunologically distinct endotypes characterized by coordinated alterations in humoral and cellular immune responses. Building on these observations, our study offers clinical evidence at the endotype level that probable type IIb CSU differs from other CSU endotypes in its cytokine expression profile, while remaining biologically interconnected with them.

IL-6 is a multifunctional cytokine involved in inflammation and autoimmunity, mediating its effects primarily through the JAK/STAT and MAPK signaling pathways.^34^^,^^35^ Although most studies report elevated IL-6 in CSU,^36^ our finding of lower IL-6 in probable type IIb CSU may reflect an endotype-specific pattern. Low IL-6 levels may reflect an immunoregulatory state associated with autoimmunity, potentially resulting from negative feedback or pathway adaptation during chronic immune activation, such as SOCS3-mediated suppression of IL-6 signaling.^37^^,^^38^ The elevated CRP in these patients is not necessarily contradictory: the discrepancy may be partly explained by the much shorter half-life of IL-6 than of CRP, and by the fact that TNF-α and IL-17 A, both elevated in this group, can synergistically enhance hepatic CRP production via IL-6-dependent and independent pathways,^39^ and other non-cytokine stimuli may also contribute. The consistent association of IL-6 with ASST positivity, TPOAb/TgAb, and reduced total IgE further supports a contextual role in type-specific immune balance rather than independent diagnostic value.

In parallel, elevated IL-17 A and TNF-α levels in probable type IIb CSU point toward enhanced Th17-driven and proinflammatory pathways. TNF-α expression is significantly elevated in both lesional and non-lesional skin of CSU patients compared to healthy controls, and serum TNF-α levels correlate positively with disease severity^40^, ^41^, ^42^, ^43^. IL-17 A is a key effector cytokine involved in tissue inflammation and immune-mediated damage,^44^ and increased Th17 cell frequencies together with elevated IL-17 A and TNF-α have been reported in ASST-positive CSU patients.^45^^,^^46^ Th1/Th17-associated cytokines (including IFN-γ and IL-21) suppress IgE synthesis, thereby establishing a rational immunological framework linking Th17 skewing to the low IgE phenotype characteristic of type IIb CSU.^47^ Consistent with this concept, our findings indicate that elevated levels of IL-17 A and TNF-α reflect a coordinated immune environment that preferentially promotes autoantibody-mediated mast cell activation rather than IgE-driven mechanisms.

Our team's previous study in CSU revealed heterogeneous inflammatory pathways and suggested IL-6 and TNF-α as candidate therapeutic targets.^48^ The potential clinical relevance of these cytokine patterns is supported by reports of clinical benefit in selected patients with refractory CSU treated with IL-17 A or TNF-α–targeted therapies.^43^^,^^49^^,^^50^ Although these observations remain limited to small-scale studies, they underscore the role of cytokine-driven immune pathways in severe and refractory disease subtypes.

Collectively, these findings suggest that cytokine expression patterns in CSU reflect coordinated immune regulation rather than isolated inflammatory signals. Alterations in IL-6, IL-17 A, and TNF-α may complement readily available clinical and laboratory parameters to support endotype-oriented stratification and inform more individualized therapeutic approaches in CSU.

### Reevaluation of nomogram prediction models

To further support individualized risk assessment, a nomogram prediction model was developed by integrating established clinical features of type IIb CSU with cytokine parameters observed in this study. The model demonstrated good predictive performance and calibration, providing a visual and quantitative tool for estimating the probability of type IIb CSU. Notably, the model reinforced the discriminative value of the 3 selected biomarkers, highlighting their complementary role in endotype-oriented evaluation.

### Limitations of the study

Several limitations should be acknowledged. First, the relatively limited sample size may affect the generalizability of our findings. Second, given the absence of a universally feasible gold standard for CSU endotyping, reliance on alternative clinical and laboratory indicators is necessary. At present, identification of type IIb CSU is best interpreted as probable, and future studies should be conducted with broader immunological assessments. Third, cytokine findings and model performance require further validation in larger, longitudinal cohorts.

## Conclusion

In summary, combinations of readily available biomarkers can provide practical references for identifying probable type IIb CSU in routine clinical practice. Among the evaluated strategies, high IgG-anti-TPO combined with ASST positivity showed the highest reference value, while high IgG-anti-TPO with low total IgE offered the greatest clinical feasibility. Distinct cytokine patterns further support immunological differences between CSU endotypes and may inform future targeted therapies. The proposed nomogram model offers an additional tool for individualized endotype assessment and clinical decision-making.

## Authorship

Jiang Ji coordinated the study and was involved in data analysis and interpretation.

Lu Chen has contributed substantially to the conception and design and was involved in drafting the manuscript.

Ni Ma, Yuxu Yao and Chuqiao Zhang were involved in data acquisition and analysis.

Enze Li was involved in study design and data analysis.

Qingqing Jiao was the overall study coordinator and was involved in proofreading the manuscript.

## Data statement

The original contributions presented in the study are included in the article. More derived data supporting the findings of this study are available from the corresponding author Jiao Q on request.

## Disclosure of the use of generative AI

During the preparation of this work, the authors used generative AI and/or AI-assisted tools solely to improve the readability and language of the manuscript.

## Disclosure of potential conflict of interest

The authors declare that the research was conducted in the absence of any commercial or financial relationships that could be construed as a potential conflict of interest.

## Figures and Tables

**Supplementary Fig. 1:**
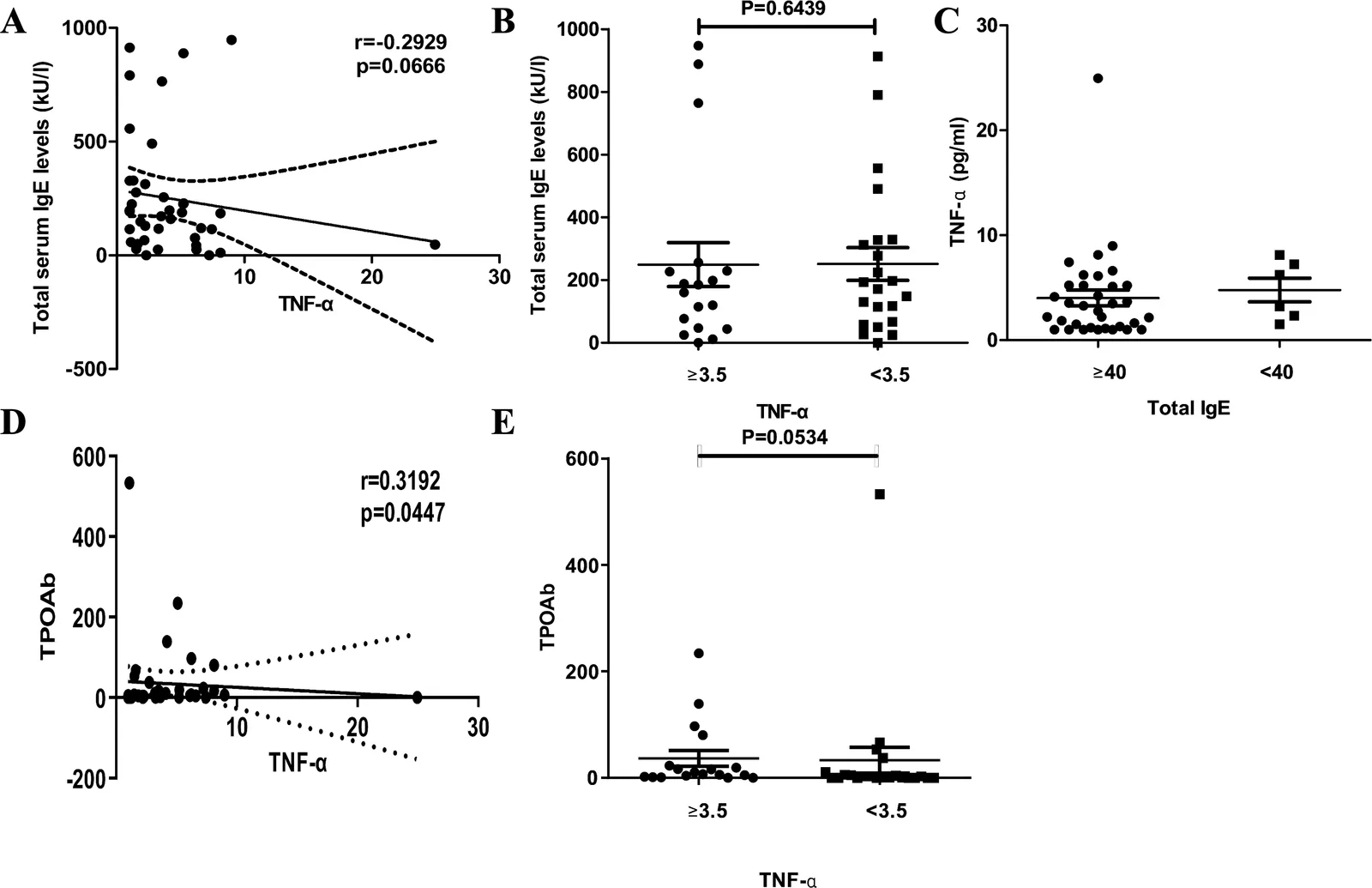
Association between serum TNF-α level and TPOAb level in CSU patients
